# A List-Based Parallel Bacterial Foraging Algorithm for the Multiple Sequence Alignment Problem

**DOI:** 10.3390/biomimetics10080485

**Published:** 2025-07-23

**Authors:** Ernesto Rios-Willars, María Magdalena Delabra-Salinas, Alfredo Reyes-Acosta

**Affiliations:** 1 Faculty of Systems, The Autonomous University of Coahuila, Saltillo 25000, Mexico; riose@uadec.edu.mx (E.R.-W.); alfredoreyes@uadec.edu.mx (A.R.-A.); 2 Faculty of Nursing, The Autonomous University of Coahuila, Saltillo 25000, Mexico

**Keywords:** BFOA, MSA, bacterial foraging algorithm

## Abstract

A parallel bacterial foraging algorithm was developed for the multiple sequence alignment problem. Four sets of homologous genetic and protein sequences related to Alzheimer’s disease among various species were collected from the NCBI database for convergence analysis and performance comparison. The main question was the following: is the bacterial foraging algorithm suitable for the multiple sequence alignment problem? Three versions of the algorithm were contrasted by performing a *t*-test and Mann–Whitney test based on the results of a 30-run scheme, focusing on fitness, execution time, and the number of function evaluations as performance metrics. Additionally, we conducted a performance comparison of the developed algorithm with the well-known Genetic Algorithm. The results demonstrated the consistent efficiency of the bacterial foraging algorithm, while the version of the algorithm based on gap deletion presented an increased number of function evaluations and excessive execution time. Overall, the first version of the developed algorithm was found to outperform the second version, based on its efficiency. Finally, we found that the third bacterial foraging algorithm version outperformed the Genetic Algorithm in the third phase of the experiment. The sequence sets, the algorithm’s Python 3.12 code and pseudocode, the data collected from the executions, and a GIF animation of the convergence on various different sets are available for download.

## 1. Introduction

Multiple sequence alignment (MSA) is a fundamental pillar in bioinformatics, which allows for the comparison and analysis of biological sequences (DNA, RNA, proteins) to uncover key information about their function, evolution, and molecular structure. The importance of MSA lies in its role as the foundation for essential tasks such as phylogenetic analysis, protein structure and function prediction, and searching genomic databases [1]. The bacterial foraging algorithm (Bacterial Foraging Optimization, BFOA) is a bioinspired optimization method that simulates the foraging behavior of bacteria. Its importance lies in its ability to efficiently, adaptively, and robustly solve complex optimization problems [2,3,4,5,6,7], often outperforming other nature-inspired algorithms [8]. The BFOA has been previously applied to the MSA problem through hybrid methodologies and multi-objective variations [9,10,11,12,13]. In this context, we present a novel strategy based on constructing a list of every aminoacidic or DNA pair from each alignment matrix and successive parallel BLOSUM evaluation of this list, which utilizes logarithmic scores: Each place in the evaluation matrix represents a log-odds score, which compares the probability of two amino acids aligning by homology, such as an evolutionary relationship, versus aligning by chance. This makes it possible to distinguish biologically plausible substitutions from random coincidences.

The main steps in the BFOA are as follows [14]:

Chemotaxis: Bacteria move through the search space by alternating between random and straight-line movements, following gradients to explore and exploit the environment. Swarming: Bacteria communicate and form groups, which helps to guide the population toward promising regions in the search space. Reproduction: After several chemotaxis steps, the best-performing bacteria reproduce, while the worst-performing ones are removed, thereby maintaining a constant population size. Elimination and Dispersal: Randomly, some bacteria are eliminated and dispersed to new locations, which helps to avoid getting stuck in local optima and maintains diversity.

These steps are repeated in cycles, allowing the algorithm to explore the solution space efficiently and find optimal (or near-optimal) solutions. This algorithm has been improved and adapted for various applications, such as feature selection, scheduling, and image segmentation, through introducing adaptive step sizes, enhanced swarming mechanisms, and even hybrid strategies [15]. It should be noted that tuning the BFOA process before any hybridization is essential. In terms of efficiency, adequate hardware and software resources are necessary when running optimization algorithms, as these resources directly impact the algorithm’s efficiency, speed, and ability to solve complex problems. The availability and type of hardware influence the performance, scalability, and accuracy of optimization algorithms [16]. In this context, parallelism is significant when running optimization algorithms as it allows multiple computations to be performed simultaneously, leading to substantial improvements in speed, efficiency, and the ability to solve larger or more complex problems. Leveraging parallelism can drastically reduce the time required for computation and enhance the quality of solutions in optimization tasks [17,18].

As stated above, MSA is an essential problem that is still under research. It is a computationally complex problem, as the number of possible alignments grows exponentially with the number and length of the sequences. This makes an exhaustive search for the best solution unfeasible [19] as sequences may present insertions, deletions, mutations, and highly divergent regions, making it challenging to identify homologous positions and obtain accurate alignments, especially among distant sequences [20]. There are two main strategies to tackle this problem: progressive pairwise alignment [21] and complete multiple alignment of the sequence matrix. In this work, we implement the second option as it provides more transparency regarding the algorithm’s capabilities in the considered task. Regarding the complexity of MSA, especially when using the sum-of-pairs (SP) scoring method, it is NP-complete. This means that finding the exact optimal alignment is computationally intractable as the number of sequences increases [22]. The MSA problem can be described as follows:

Given a set of *n* sequences *S = {s_1_*, *s_2_*, *...*, *sₙ}*, where each sequence *sᵢ* has a length *lᵢ*, the goal is to find an alignment that maximizes the similarity among all sequences simultaneously. The alignment can be represented as a matrix A of dimensions *n × m*, where *m* is the length of the final alignment, and each element *aᵢⱼ* can be a character from the genetic alphabet Adenine, Cytosine, Guanine, and Thymine (A, C, G, T) or a gap (-), mathematically represented as *ε.*

The objective function typically seeks to maximize the following:

*Score_total = Σᵢ₌_1_^n−1^ Σⱼ₌ᵢ₊_1_ⁿ Σₖ₌_1_ᵐ w(aᵢₖ, aⱼₖ)* (1)

where *w(aᵢₖ*, *aⱼₖ)* is the scoring function for aligned characters, and *k* represents the position in the alignment.

The scoring function generally considers the following:

1. Matches: *w(x*,*x) = α* (positive value);
2. Mismatches: *w(x*,*y) = β* (negative value);
3. Gaps: *w(x*,*ε) = w(ε*,*x) = γ* (penalty).

The problem’s computational complexity is *O(lⁿ)*, where l is the average length of the sequences. This problem can also be formulated in terms of the minimization of the sum-of-pairs (SP) score:

*SP_score = min Σᵢ₌_1_^n−1^ Σⱼ₌ᵢ₊_1_ⁿ d(sᵢ*, *sⱼ)* (2)

where *d(sᵢ*, *sⱼ)* is the distance between each pair of aligned sequences.

As stated before, this problem is NP-complete, meaning that there is no efficient algorithm which is guaranteed to find the optimal solution for an arbitrary number of sequences.

A hypothetical alignment matrix of four genetic sequences could have three alignment cases: match, mismatch, and gap.

## 2. Materials and Methods

As described above, the BFOA implements four optimization steps. Accordingly, the interaction of bacteria is expressed as a function g():

(3) $$\begin{array}{ll} g\left({cell}_{k}\right) & =\sum_{i=1}^{S}\left[{-d}_{attr}\;*\;e^{\left({-w}_{attr}*\sum_{m=1}^{P}{\left({cell}_{m}^{k}-{other}_{m}^{i}\right)}^{2}\right)}\right]\\ & +\sum_{i=1}^{S}\left[h_{repel}\;*\;e^{{\left({-w}_{repel}*\sum_{m=1}^{P}({cell}_{m}^{k}-{other}_{m}^{i}\right)}^{2}}\right] \end{array}$$

where *cell* denotes the bacterium at the time of the evaluation iteration, and *other* refers to a neighboring cell. The parameters *d_attr_* and *w_attr_* are coefficients of attraction, and the parameters *h_repel_* and *w_repel_* are related to repulsion. Finally, *S* is the number of cells in the population, and *P* is the number of dimensions in the optimization problem. Appendix A provides the pseudocode for a general implementation of the BFOA.

### 2.1. The Developed BFOA

In this work, we developed a BFOA using the Python programming language. The main advantage of this strategy is the parallelization of the main cycles in the optimization process. The algorithm starts the main class in the *parallel_BFOA.py* document; it starts the execution by importing the other necessary classes in the different documents, in addition to the *multiprocessing*, *numpy*, and *copy* libraries. Once all the tools used in this class have been imported, the variables indicating the number of bacteria that will make up the initial population, the number of iterations of the optimization cycle, and the range of tumbling are initialized. A list containing the genetic sequences that represent the bacteria is initialized. Next, the *fastaReader* class is used, which is responsible for abstracting the genetic sequences and their names, which are later saved in variables that are finally passed to the main class. As the genetic sequences and their names are stored in a FASTA file, it is necessary to pass the relative or global path of said document.

The variables *d_attr_*, *w_attr_*, *h_repel_*, and *w_repel_* are initialized. The Manager class is then referenced by saving it in a variable, creating a multiprocessing instance. *Manager*() is an object in the multiprocessing library for parallel processing. The number of sequences that will be aligned and printed on the console is saved in a variable. To start the parallel multithreading process, a list containing a shared list is created using the manager.list() method, which can be modified by multiple processes in parallel, as well as another shared list with the names of the streams. Finally, a shared list called NFE is initialized, containing the evaluation results for the bacteria.

Two methods are created: One initializes the population of bacteria, in which the genetic sequences are loaded into a list named *bacterium* that is restarted in each cycle according to the number of iterations previously defined; however, before that, it is added to an array called a population that contains all the new lists of bacteria. The second method is printPopulation which, as its name indicates, helps to print the sequences of the bacteria in the population on the console. Lists shared between parallel processes are initialized. Each list represents a table of bacterial model data:

- blosumScore: Alignment scores according to the BLOSUM.
- tablaAtract, tablaRepel: Forces of attraction and repulsion between bacteria.
- tablaInteraction: Sum of *Attract* and *Repel* tables.
- tablaFitness: Total evaluation for each bacterium.
- granListaPares: List of amino acid pairs in each alignment.
- NFE: List of amino acid pairs in each alignment.

The resetLists method, as its name implies, re-initializes all shared lists and creates a new manager. It is used in each algorithm iteration to prevent the accumulation of old data. The following method in the class is the *cuadra* method, which ensures that all sequences have the same length, filling the shorter ones with “-” (gaps) to avoid future indexing errors in subsequent operations. The “*column-clean”* method goes through the sequence matrix and eliminates the columns formed of only gaps, relying on the “*gapCulmn*” (which detects this type of column) and “*deleteCulmn*” (which removes the column) methods.

**Figure 1:**
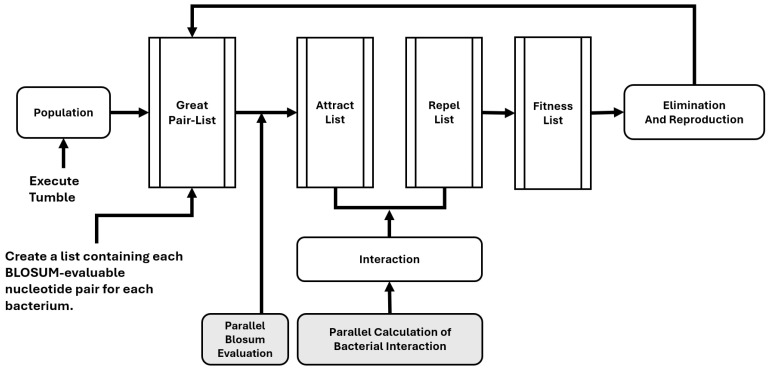
Scheme of the developed algorithm that highlights the parallelized parts of the process.

The class also has methods for the evaluation of the bacteria, supported by two methods and the class *evaluadorBlosum*, which assesses the row and returns a grade based on the alignment and number of gaps in the sequences. The BLOcks SUbstitution Matrix (BLOSUM) refers to a family of amino acid substitution matrices derived from conserved local alignments (“building blocks”) of related proteins, without assuming an explicit evolutionary model [23]. In this work, we implement the widely used BLOSUM62. In the *creaTablasAtractRepel* method, the threads from *ThreadPoolExecutor* are used to calculate the attraction and repulsion in parallel, resulting in a score. With this score, using the “*creaTablaFitness*” method, a final score can be calculated for each bacterium by adding the score calculated using *evaluatorBlosum* and its interactions with other bacteria. In the end, the best bacteria (evaluated with the *getBest* method) are selected, while the worst ones are eliminated (with the *replaceWorst* method) and are replaced with copies of the bacteria that were best evaluated. Figure 1 presents a diagram of the developed algorithm. The primary strategy for parallelization is using lists as “notebooks” where the parallel process can write simultaneously, with each register corresponding to a different bacterium from the population. We parallelized tasks in Python using *multiprocessing.Pool()*, which significantly improves computational efficiency by distributing workloads across the CPU cores, allowing for a reduction in execution time. Due to its scalability, it is particularly advantageous for computationally intensive applications, including large-scale data processing and modeling. The Python code is available for download. As described above, constructing a list of evaluable pairs is the main characteristic of this BFOA approach. In this way, the algorithm builds a list of every amino acid pair from each column in the matrix for a given set of sequences to be aligned. Then, the BLOSUM evaluation is performed on the parallel resources. This approach provides a more rapid evaluation and successive chemotaxis process, as the BLOSUM evaluation proceeds simultaneously with the other processes in the alignment matrix.

In a hypothetical scenario designed to illustrate the computational demands of multiple sequence alignment, we considered the case of 100 alignment matrices, each comprising 100 sequences of 100 amino acids in length. All unique pairs of amino acids must be evaluated using the BLOSUM for each column within these matrices. The binomial coefficient C(100, 2) gives the number of unique pairs per column, resulting in 4950 pairwise comparisons for each column. Given that each matrix contains 100 columns, this leads to 495,000 comparisons per matrix. When extended to 100 such matrices, the total number of pairwise evaluations required reaches 49,500,000. Supposing a hypothetical evaluation time of 1 millisecond per pairwise comparison, under a sequential processing regime, the total computation time for all matrices would amount to approximately 13.75 h. However, the adoption of parallel processing strategies can dramatically reduce this time. For instance, utilizing 16 processing cores would decrease the total computation time to approximately 52 min, while employing 128 cores reduces it to just 7 min. This linear scaling demonstrates the substantial efficiency gains which are achievable through parallelization. However, in practice, the number of iterations and the successive alignment evaluation after each mutation influence the processing time. Finally, with the evaluation results, the attraction and repulsion lists (capturing the interaction process) are built in parallel for the fitness calculation.

### 2.2. Genetic Sequences

The genetic sequences used in this work were downloaded from the NCBI [24] database. All genetic sequences are related to human health, particularly that in older persons. Appendix B outlines the four sets of genetic sequences: set A is described in Appendix B.1, the set B is described in Appendix B.2, the set C in Appendix B.3, and the set D in Appendix B.4. Additionally, a detailed length comparison is provided in Table A1, Table A2, Table A3 and Table A4. 

### 2.3. Performance Evaluation Metrics

The metrics of interest for the purposes of performance comparison were as follows: (1) The *fitness* is a numeric representation of a bacterium’s capacity to efficiently reach better solutions to the alignment problem. It is essential to point out that Equation (3) describes the fitness calculation. The other evaluation metrics include (2) the processing time (*t*), which is widely used as a comparison metric among optimization algorithms, and (3) the number of function evaluations (*NFE*), which is the number of times that the functions must be evaluated by the algorithm to reach the corresponding result; more specifically, the algorithm counts the calculation of each attraction or repulsion among the bacteria in the population. In the case of finding an indel (i.e., a pair of characters where one of them is a gap), the algorithm imposed a penalty of 0.1 on the BLOSUM evaluation process.

### 2.4. Hardware Resources

Every test run of the algorithm developed in this work was performed using a multi-core supercomputer with 1 Tbyte storage for hosting data, 128 cores divided into four nodes, and 192 GB of RAM.

### 2.5. Algorithm Initialization

The population is a sensitive parameter that is critical in the optimization process. The number of bacteria in the set of schemes varies from 8 to 120, depending on the given scheme. Also, the tumbling process is defined as the number of gap insertions. The initialization of this parameter varies from 100 to 330, also depending on the given scheme. Furthermore, the BFOA implements the attraction and repulsion parameters. In this work, the initialization involved varying the *w_rep_* parameter from 0.001 to 0.002, and in the final phase, it was set to 1. Regarding the alignment matrix, the initialization consists of the insertion of gaps corresponding to the tumble parameter. All the parameters are initialized in each of the 30 algorithm runs. Generally, each scheme represents a distinct initialization scenario. 

### 2.6. Experiment

To explore the performance of the BFOA, we designed a three-phase experiment. The first phase involved an empirical exploration based on schemes of BFOA parameters, where the first approach provided data from one alignment made under each scheme for the genetic sequences from Set A. Subsequently, in the second phase, we used the rest of the genetic and/or protein sequences to perform alignments in a 30-run scheme. Finally, the third phase was configured taking into consideration the results from the first and second phases. The details of each phase are described below.

In the first phase, we tested the performance of the BFOA and the developed parallel process using Set A of sequences to perform alignment and compare the algorithm’s results. For this purpose, we established an experiment to compare the values of the previously described evaluation metrics. We configured the 4 test schemes described in Table 1, where the algorithm’s parameters were changed at different levels to determine those with which the algorithm performed best. It is worth noting that each scheme (from A to D) presents differing tumbling, bacteria, and iteration settings while maintaining identical attraction and repulsion parameters. With this strategy, we expected to observe differences in performance regarding the parameters mentioned, which are known to relate directly to the computational effort. After that, scheme K was incorporated, as described below.

In the second phase, we chose new parameters based on the results from the first phase. In particular, we changed (a) the *w_rep_* parameter from 0.001 to 0.002 to increase the strength with which bacteria repel each other; (b) the population size to 120 bacteria and the tumbles to 200; and (c) the number of iterations to 33 during optimization. This set of parameters was named scheme K. We also included the *swim* process in the algorithm, which consists of deleting random gaps to avoid sequences with gap saturation. For clarity, we named this algorithm BFOAd (for the gap deletion process). Finally, we incorporated three sets of sequences for performance comparison among the BFOA vs. BFOAd, each in a 30-run process. Table 2 details the second phase of the experiment. The number of random gaps deleted in the BFOAd was 50.

The comparison consisted of the statistical discrimination of mean values using the Mann–Whitney U test for cases with atypical values and a non-normal distribution, while the Shapiro–Wilk test was used to determine those with a normal distribution. Additionally, a *t*-test was performed to compare differences. Then, the Cohen test was carried out to assess the effect size.

Finally, the third phase included the development of the well-known Genetic Algorithm for performance comparison. Additionally, the bacterial foraging algorithm was modified into a new version called BFOAdtp, which primarily incorporated an elitism operator to enhance fitness.

## 3. Results

As previously described, the results correspond to the three-phase experiment, with the first phase exploring the algorithm’s performance and the second phase involving 30 runs, with the parameters having been empirically adjusted based on the results obtained in the first phase.

### 3.1. First Phase

Presenting the results of the experiment’s first phase, Table 3 displays the best fitness value achieved by the algorithm, as well as the NFE and time in seconds (t). Scheme D performed the best in terms of fitness value; however, it sacrificed time and had an increased NFE value. As described above, the higher the fitness value, the better.

**Figure 2:**
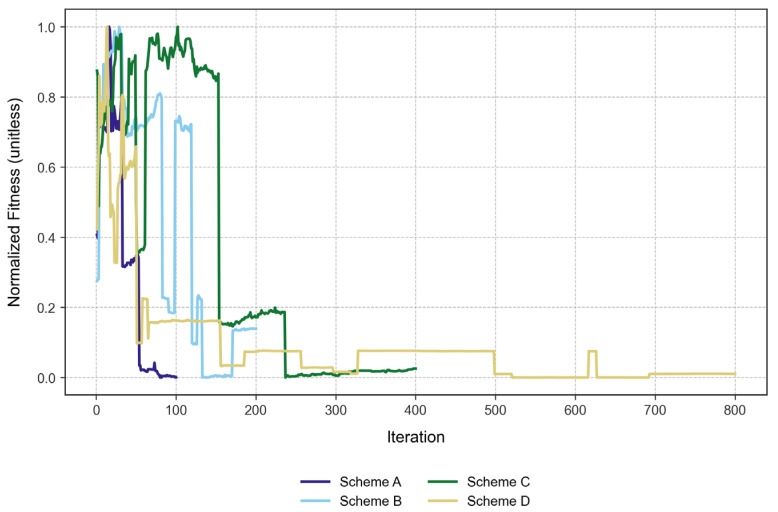
The normalized fitness value from each scheme, showing the poor performance of the BFOA for all schemes after approximately iteration 150.

On the other hand, we found that the solutions obtained with the algorithm tended to worsen from an iteration number of approximately 150. As a representation of this occurrence, we normalized the fitness values from each scheme and obtained Figure 2.

This observation may provide justification for stopping the algorithm before its fitness value starts to worsen, as the genetic sequences may reach a gap-saturated status since the algorithm reports the best bacteria from the given iteration without elitism. This process permits the worsening of the solutions through iterations and serves as an exploration of the algorithm’s performance.

In terms of convergence, we found that scheme D converged the fastest, reaching 95% of its best fitness within just 13 iterations. This is interesting, as Scheme D also had the most demanding parameters (i.e., more bacteria, more iterations), but it found reasonable solutions quickly. Scheme A reached 95% of its best fitness within 16 iterations, showing rapid convergence despite being the simplest scheme. Finally, scheme B took 22 iterations, while scheme C took 25 to reach the same threshold. As such, these two schemes converged more slowly than schemes A and D.

The convergence speed does not strictly increase with the complexity of the scheme. While Scheme D was the most complex and converges fastest, Scheme A (the simplest) also converged rapidly. Schemes B and C were intermediate in complexity and took longer to converge; this could be due to the larger search space or the algorithm exploring more before settling.

### 3.2. Second Phase

In this part of the work, we compared the performance of the BFOA and BFOAd with respect to the alignment of three sets of sequences (B, C, D) using scheme K and a 30-run experiment for comparison. The performance metrics were the same as in the previous phase, namely, fitness, NFE, and time (t). Table 4 details the statistical results.

The most remarkable statistical feature is the considerable variability and occasional high performance of the BFOAd on Set B. However, the BFOAd achieved better fitness values on all three sets in comparison to the BFOA. This suggests that the BFOAd may have potential for exceptional results. The standard deviation of the BFOAd in fitness values is also higher in Sets C and D, which describes the variability in the solutions found, especially in Set D. The computational time and number of function evaluations (NFE) were higher across all datasets, about 10–18% slower than the BFOA. However, more extensive swimming may also lead to a more time-consuming process.

The BLOSUM score—which measures biological alignment quality—was higher for the BFOA on all datasets, especially Set B, where the BFOAd’s score was observed to decrease dramatically. The relative performance ratios confirmed that the only dramatic difference was in fitness for Set B, where the BFOAd sometimes found much better solutions. However, this came with a loss in biological quality (i.e., BLOSUM score) and much higher variability. From a computational efficiency perspective, the BFOA and BFOAd are very similar, with the BFOAD being marginally more demanding in time but not in the number of evaluations. Table 5 describes the statistically significant differences between the BFOA and BFOAd applied to the sequence sets in terms of the fitness, time, and NFE metrics.

First, it can be observed that the BFOAd was significantly slower and required more function evaluations (NFE) than the BFOA on all datasets. The Cohen’s d values for these metrics were enormous, indicating that the difference is statistically significant and relevant in practice. Regarding fitness, the differences were small or unimportant, and, in some cases, the BFOA performed slightly better. This suggests that the BFOA is more efficient and maintains or improves the quality of solutions, while the BFOAd does not justify its higher computational cost. Additionally, the Cohen’s d values reinforce this interpretation: practical differences are essential in efficiency but negligible in solution quality. The collected data are available for download and further analysis. Finally, Table 6 presents a compact side-by-side comparison of the three key metrics of the BFOA and BFOAd. The execution time in seconds of the BFOAd is an order of magnitude longer than that of the BFOA for every dataset. The NFE of the BFOAd shows that it consistently requires 2–3× more evaluations than the BFOA to reach its final solution. The fitness is improved for the BFOAd only in Set B.

**Figure 3:**
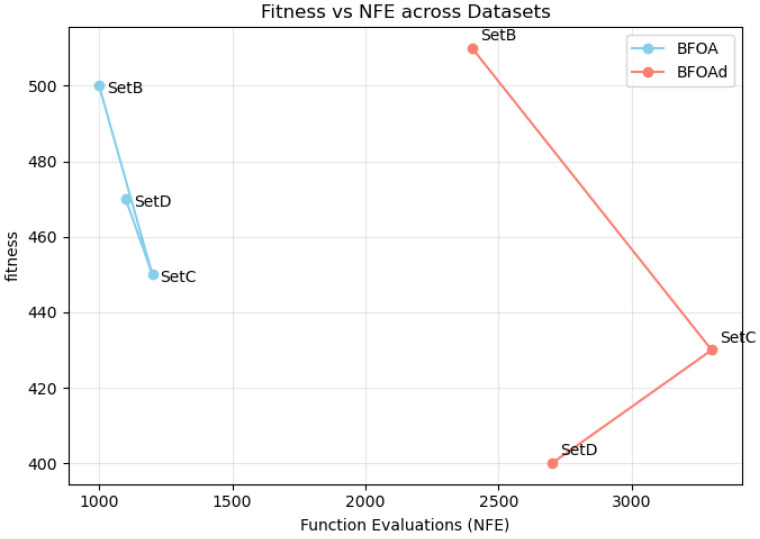
A comparison of the behaviors of the BFOA and BFOAd across all three datasets (Set B, Set C, and Set D).

As a general summary, Figure 3 compares the convergence behaviors of the BFOA and BFOAd versus the NFE, across all three datasets (i.e., Set B, Set C, and Set D) in a single panel.

Across all datasets, both algorithms showed rapid initial improvement, followed by a slower approach to their best solutions. For Set C and Set D, the BFOA generally achieved better (lower) fitness values than the BFOAd, especially in the early and middle stages of optimization. In Set B, the performance of the BFOAd was better.

### 3.3. Third Phase

We found a high variance in the fitness value from the previous phase, which may be due to the way the BFOA calculates the fitness value, considering the interaction between each bacterium and the others in the population with the BLOSUM score, as described in Equation (3). Also, the gap deletion process in the BFOAd increased the variability in the alignment matrices of the bacterial population. 

Thus, we developed a third version of the BFOA, named BFOAdtp, based on the results of the previous phases. The configured changes are as follows: (a) The BLOSUM evaluation values were normalized for greater biological significance. (b) The elitism operator was incorporated to keep the N best bacteria in the population. Also, (c) we developed an improved bacteria substitution method, where the best bacteria of each iteration were discarded if they were not better than the best bacteria of the previous iteration. And finally, (d) the *w_rep_* value was changed to 1 to increase the degree of the algorithm’s bacterial dispersion and exploration. Regarding the NFE, we updated the count process to correspond to the evaluation of each bacterium’s alignment matrix, since the BFOA and BFOAd were counting evaluation functions for each repulsion and attraction interaction.

A Genetic Algorithm (GA or AG) was developed for performance comparison. This well-known optimization method from John Holland, based on evolutionary principles, has proven effective in challenging problems like Multiple Sequence Alignment (MSA). The parameters of both algorithms and the experiment scheme are described in Table 7. 

Set B of genetic sequences was used for a performance comparison of the GA vs. BFOAdtp, with an interest in the fitness and NFE metrics.

For data clarification and performance visualization, we calculated the z-score from the fitness metric of the 30 runs. Every fitness measurement *fk*,*t* from each run becomes

(4) $$z_{k,t}=\frac{f_{k,t}-\pi_{k}}{\sigma_{k}}$$

**Figure 4:**
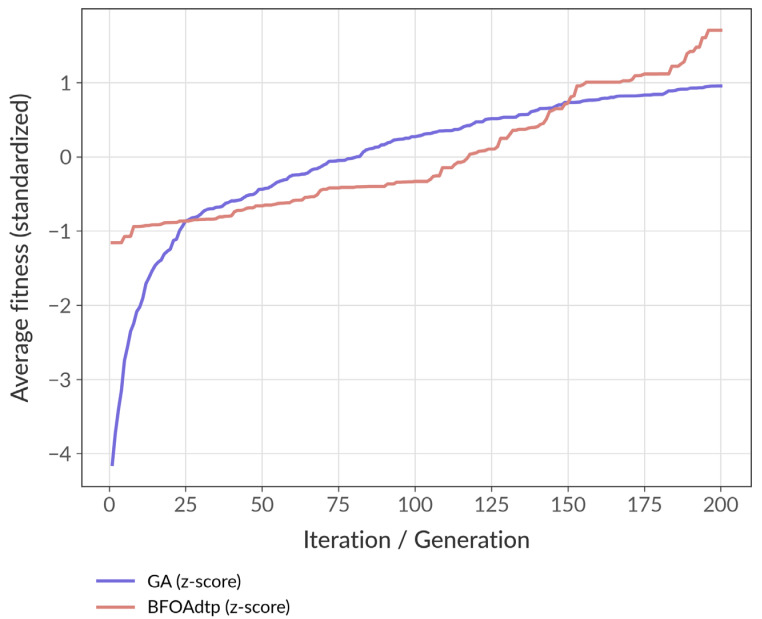
The standardized fitness values of the GA vs. BFOAdtp. The vertical axis represents the average standardized fitness (z-score): 0 = run mean; +1 = one standard deviation better.

Raw GA and BFOA fitness numbers differ in magnitude and variance. Converting each run to a z-score erases the scale differences, which enables us to overlay and average runs fairly. The curves in Figure 4 show how far, in standardized units, each algorithm climbs over time. A higher z-score is a direct measure of quality progress.

We found that in the last 50 iterations, the BFOAdtp remains approximately 0.4 σ above the GA, which means that, relative to its own variability, the BFOAdtp achieves solutions that are several tenths of a standard deviation better on average. Additionally, the GA rises quickly but plateaus near +1 σ. In contrast, the BFOAdtp continues to climb well past that point, indicating a stronger exploitation phase that persists in extracting gains after the GA has stalled. However, both algorithms start below their run means. Yet, the BFOA begins closer (≈ –0.3 σ), while the GA plunges to about –0.8 σ, considering that a smaller negative offset implies more promising initial solutions and less risk of a poor early population. Finally, we found greater run-to-run consistency because each run was standardized before averaging, and a smoother BFOAdtp trajectory indicates lower volatility across the 30 runs. The GA exhibits sharper swings in the first 40 generations, suggesting a stronger dependence on random starting conditions.

**Figure 5:**
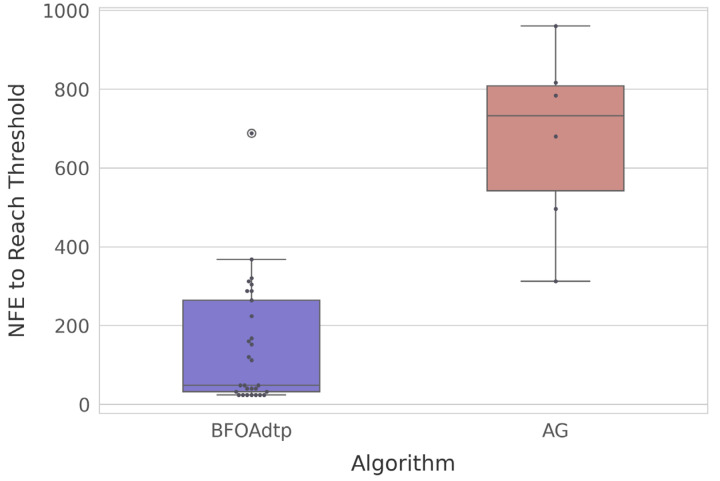
A boxplot of the NFE values required to reach the threshold for both algorithms.

Regarding the NFE metric, both algorithms record it as an accumulated counter that increases by eight evaluations per step in the BFOA and by eight in the AG (16 → 24 → 32 → …), so every run shows practically identical NFE values at the same iteration or generation index. However, the GA started with sixteen counts, while the BFOAdtp started with eight due to the first selection process. For comparison, using this metric, we took the best fitness achieved by both the AG and BFOAdtp for each run (*n* = 60). Then, the median was calculated and defined as the threshold of interest (*tHi*). Then, we discarded the runs that never reached *tHi*, that is, twenty-four from the AG and one from the BFOAdtp were discarded because they would not provide information for NFE comparison. Figure 5 shows a boxplot of the NFE values required to reach the threshold. 

Each dot represents a run that successfully reached the fitness threshold; the colored box and whiskers summarize the distribution of the NFE at the moment of success. We found that the BFOAdtp achieves the target in dramatically fewer evaluations: median NFE ≈ 80, with 75% of runs below NFE ≈ 200. Only one run fails to appear because it never hit the threshold. On the other hand, the AG manages to reach the goal in just six runs, all of which require an NFE value of between ≈approximately 300 and ≈approximately 1000. Thus, the BFOAdtp requires an order of magnitude fewer evaluations to achieve the same quality level, thereby saving computational time and energy.

## 4. Discussion

The results indicate that both the BFOA and BFOAd exhibit the expected convergence pattern, characterized by rapid initial improvements in fitness followed by a plateau as the algorithms approach their optimal solutions. Notably, the BFOA consistently demonstrated superior or comparable performance to the BFOAd across all datasets. In Set B and Set D, the BFOA achieved lower fitness values more quickly and maintained an advantage throughout optimization. In Set C, the performance of both algorithms was nearly indistinguishable, suggesting that the characteristics of this dataset may neutralize the advantages conferred by the modifications in the BFOAd. These findings indicate that the original BFOA is generally more robust and effective across diverse problem instances, particularly in the early and middle stages of optimization. The observed differences in convergence behavior may be attributed to the specific parameter settings or structural modifications introduced in the BFOAd, which could interact differently with the landscape of each dataset. For further analysis, we recommend systematically exploring key algorithm parameters such as attraction and repulsion, as well as incorporating more extensive sets of genetic sequences.

## 5. Conclusions

The present study shows that the bacterial foraging algorithm is an effective alternative for tackling the Multiple Sequence Alignment (MSA) problem. Across four Alzheimer-related datasets, the BFOAdtp achieved significantly better fitness values than its comparator variants, demonstrating better performance. However, the three developed bacterial algorithms showed improvement, as the basic BFOA provided the first approach but yielded a high NFE value and poor fitness achievement. Then, the BFOAd version incorporated a swim process that deleted gaps from the alignment matrix. Finally, the third version, BFOAdtp, incorporated elitism and showed better performance compared to the well-known Genetic Algorithm. The use of *t*-tests and Mann–Whitney tests in the second phase confirmed statistical significance. The parallel design consistently reduced the execution time while preserving alignment quality, underscoring its computational efficiency. The list-based population handling enabled scalable performance over a range of problem sizes, and the alignments are suitable for immediate biological relevance to phylogenetic analyses and drug design research. Future work will focus on hybrid affinity heuristics to accelerate chemotaxis, evaluating the algorithm on larger metagenomic datasets, extending parallelization to distributed computing environments, and developing a sequence preprocessing stage that detects conserved domains in the alignment matrix to yield biologically richer results. Additionally, the fitness, NFE, and time showed a contrasting panorama.

## Figures and Tables

**Table 1:** Parameter values in the different schemes.

Parameter	Scheme A	Scheme B	Scheme C	Scheme D	Scheme K
Tumble	100	160	180	330	200
Number of Bacteria	22	44	88	120	120
Iterations	100	200	400	800	33
*d* *_attr_*	0.1	0.1	0.1	0.1	0.1
*w* *_attr_*	0.002	0.002	0.002	0.002	0.002
*h* *_rep_*	0.1	0.1	0.1	0.1	0.1
*w* *_rep_*	0.001	0.001	0.001	0.001	0.002
Swim	0	0	0	0	50

**Table 2:** Description of the second phase of the experiment in a 30-run process.

Algorithms	Scheme	Set
BFOA vs. BFOAd	K	B
BFOA vs. BFOAd	K	C
BFOA vs. BFOAd	K	D

**Table 3:** Results of the experiment’s first phase.

Parameter	Scheme A	Scheme B	Scheme C	Scheme D
Best Fitness	52.0	77.7	124.9	185.7
NFE	95.4 K	866.9 K	6.9 M	26.6 M
t	1.7 K	8.8 K	52.9 K	292.8 K

**Table 4:** Descriptive statistics obtained in phase 2 of the experiment. The fitness, BLOSUM score, interaction, time, and NFE are presented as mean values with their corresponding standard deviation values.

Algorithm	Dataset	Fitness	BLOSUM Score	Interaction	Time	NFE
BFOA	Set B	1648.01 ± 3326.64	23.92 ± 28.34	1624.09 ± 3329.26	6.2 K ± 8.98	537.0 K ± 300.4 K
BFOAd	Set B	43,026.36 ± 234,031.43	0.18 ± 32.54	43,026.19 ± 234,033.01	7.3 K ± 12.03	540.3 K ± 302.8 K
BFOA	Set C	45.80 ± 2.59	14.53 ± 2.90	31.28 ± 3.46	6.0 K ± 22.89	542.0 K ± 303.1 K
BFOAd	Set C	46.94 ± 3.78	13.76 ± 4.49	33.18 ± 5.72	6.8 K ± 6.59	549.2 K ± 307.5 K
BFOA	Set D	50.51 ± 18.69	11.84 ± 7.43	38.67 ± 20.29	6.1 K ± 6.39	545.3 K ± 305.2 K
BFOAd	Set D	58.67 ± 74.17	8.46 ± 9.65	50.21 ± 80.30	6.7 K ± 7.85	551.7 K ± 308.8 K

**Table 5:** Description of the significant differences in time, NFE, and fitness values.

Dataset	Metric	BFOA	BFOAd	Test	*P* value	Significance	Cohen’s d
SetB	Time (s)	104,255.7 ± 164.8	117,654.2 ± 185.2	*t*-test	6.47 × 10^−94^	(*p* < 0.001)	76.438
SetB	NFE	1040.3 K ± 6.6 K	1048.9 K ± 5.7 K	Mann–Whitney U	4.57 × 10^−9^	(*p* < 0.001)	1.397
SetB	Fitness	1194.78 ± 1886.0	1713.25 ± 3358.5	Mann–Whitney U	0.0657	ns	0.19
SetC	Time (s)	101,631.5 ± 534.7	110,419.7 ± 102.5	*t*-test	1.48 × 10^−63^	(*p* < 0.001)	22.827
SetC	NFE	1049.6 K ± 6.7 K	1065.0 K ± 4.6 K	Mann–Whitney U	5.07 × 10^−10^	(*p* < 0.001)	2.679
SetC	Fitness	47.65 ± 2.02	46.71 ± 5.17	Mann–Whitney U	7.30 × 10^−4^	(*p* < 0.001)	−0.238
SetD	Time (s)	102,564.4 ± 140.1	109,511.8 ± 154.4	Mann–Whitney U	3.02 × 10^−11^	(*p* < 0.001)	47.113
SetD	NFE	1056.5 K ± 6.6 K	1069.8 K ± 4.7 K	Mann–Whitney U	5.07 × 10^−10^	(*p* < 0.001)	2.313
SetD	Fitness	36.87 ± 5.16	32.88 ± 3.94	Mann–Whitney U	0.1297	ns	−0.867

**Table 6:** A description of the NFE, fitness, and time for the BFOA and BFOAd.

Dataset	BFOA Time	BFOAd Time	BFOA NFE	BFOAd NFE	BFOA Fitness	BFOAd Fitness
SetB	1282.2	11,213.2	1000	2400	500	510
SetC	1282.2	15,713.2	1200	3300	450	430
SetD	1282.2	18,913.2	1100	2700	470	400

**Table 7:** Description of the GA and BFOAdtp in the third phase.

Algorithm	Population	Iterations/Generations	Exploration Process	Parameters
BFOAdtp	8	200	Tumble = 100, swim = 1	*d_attr_ = 0.1 w_attr_ = 0.002 h_repel_ = d_attr_ w_repel_* = 1
GA	8	200	Roulette selection and Two-point Crossover	Mutation Probability = 0.3

## Data Availability

The original data presented in the study are openly available in the Supplementary Materials.

## Supplementary Materials

The following supporting information can be downloaded: Sets of genetic sequences, https://usegalaxy.org/u/rioswillars/h/bfoa-algorithm-tests (accessed on 16 July 2025). A BFOA animation, https://usegalaxy.org/u/rioswillars/h/bfoa-anim-msa (accessed on 16 July 2025). Parallel BFOAdtp, https://github.com/riosew/BFO-Algorithm-MSA (accessed on 16 July 2025).

## Abbreviations

The following abbreviations are used in this manuscript:

BFOA	Bacterial Foraging Algorithm
MSA	Multiple Sequence Alignment

## Appendix A

Bacterial Foraging Optimization Algorithm (BFOA) pseudocode.

**Algorithm A1:** Bacterial_Foraging_Optimization
// Initial Parameters
p = number of bacteria in the population
S = number of dimensions of the search space
Nc = number of chemotactic steps
Ns = number of swim steps
Nre = number of reproduction steps
Ned = number of elimination-dispersal events
Ped = probability of elimination-dispersal
C = step size

// Initialization
For each bacterium i from 1 to p:
Initialize random position Xi in the search space S
Calculate fitness J(Xi)

// Main Loop
For l = 1 to Ned:
For k = 1 to Nre:
For j = 1 to Nc:
For i = 1 to p:
// Chemotaxis
Generate random direction Δ(i)
// Movement
Xnew = Xi + C ∗ Δ(i)
Calculate J(Xnew)

// Swimming
m = 0
While m < Ns:
If J(Xnew) < J(Xi):
Xi = Xnew
Xnew = Xi + C ∗ Δ(i)
Calculate J(Xnew)
m = m + 1
Else:
m = Ns
End While
End For

// Reproduction
If k == Nre:
Sort bacteria by fitness value
Remove half of the bacteria with worst fitness
Duplicate the half with best fitness
End If

// Elimination-dispersal
For each bacterium i:
If random(0,1) < Ped:
Relocate bacterium i to a random position
End If
End For
End For
End For
End For
Return the best solution found
End Algorithm

## Appendix B

The sets of sequences used in this work are described in this appendix. For this purpose, each set integrates different genetic and protein sequences.

### Appendix B.1. Set A

Set A of sequences is made up of four elements that are closely related to Alzheimer’s disease: The sequence NM_000484.4 corresponds to Homo sapiens and encodes the amyloid beta precursor protein (APP), specifically, transcription variant 1. This protein plays a crucial role in brain development and is directly implicated in Alzheimer’s disease [25]. Additionally, sequence NM_001198823.1 belongs to Mus musculus (the mouse) and also encodes the amyloid beta precursor protein in its transcription variant 1. The mouse is a fundamental model for studying the function of APP in mammals, as it shares many functional similarities with the human version [26]. In the case of Drosophila melanogaster, sequence NM_001259804.2 corresponds to a gene called “approximated” (app), in its transcription variant S. This gene is the homolog of APP in the fruit fly and is essential for evolutionary studies, as it allows for the comparison of the conservation and functional divergence of this gene throughout evolution. Finally, sequence AF389401.1 belongs to Danio rerio (the zebrafish) and represents the amyloid precursor protein in its complete coding sequence (CDS). The zebrafish is an important model for research on development and neurodegenerative diseases. Together, these sequences represent the same gene—namely, APP—in different species. This is particularly valuable for evolutionary studies of the APP protein, research on Alzheimer’s disease, understanding the conserved function of this protein in other organisms, and the development of animal models for biomedical research. Table A1 provides a description of the basic statistics of Set A.

**Table A1:** Description of the four genetic sequences that comprise Set A.

Species (ID)	Length (Nucleotides)	GC Content	A (%)	T (%)	C (%)	G (%)
NM_000484.4	3583	48.5%	27.2%	24.3%	22.5%	26.0%
NM_001198823.1	3377	50.9%	27.1%	22.0%	24.6%	26.2%
NM_001259804.2	2679	47.1%	28.2%	24.6%	21.6%	25.5%
AF389401.1	2217	58.5%	23.5%	18.0%	26.3%	32.3%

### Appendix B.2. Set B

ABCA7 proteins in humans and other mammals comprise Set B of sequences. The ABCA7 protein has important functions related to the health of older adults, such as lipid transport; in particular, it is a phospholipid-transporting ATPase that plays a crucial role in the metabolism and transport of lipids in cells. It has a relationship with Alzheimer’s, as scientific studies have shown that ABCA7 is significantly related to Alzheimer’s disease because it aids in the removal of beta-amyloid peptide [27]. As such, mutations in this gene may increase the risk of developing Alzheimer’s [28]. It also participates in the processing of amyloid precursor protein (APP) [29]. Table A2 details the basic characteristics of the sequences in Set B.

**Table A2:** Description of the protein sequences in Set B.

Accession_ID	Species	Length	Polar_%	Charged_%	Hydrophobic_%
NP_061985.2	Homo sapiens	2146	21.06	21.3	57.64
XP_044943956.1	Mustela putorius furo	1820	20.33	20.27	59.4
XP_063575887.1	Pongo abelii	1811	21.31	20.38	58.31
XP_070321384.1	Odocoileus virginianus	1710	21.64	19.42	58.95
XP_053906274.1	Cuculus canorus	2244	23.48	20.37	56.15

### Appendix B.3. Set C

EphA1 is a member of the Eph receptor tyrosine kinase family, which plays a crucial role in cell signaling, tissue organization, and cellular communication. It is highly conserved among mammals, meaning that its function is similar across several species. In the context of the health of older adults, EphA1 has several important implications; for example, its dysregulation has been linked to neurodegenerative diseases such as Alzheimer’s disease [30]. EphA1 is also overexpressed in Parkinson’s models [31]. Table A3 details the sequences in Set C.

**Table A3:** Descriptive characteristics of the sequences in Set C.

Accession_ID	Species	Length	Polar_%	Charged_%	Hydrophobic_%
NP_005219.2	Human	976	25.61	23.67	41.6
NP_034258.2	Mouse	977	26.92	23.23	41.04
XP_016803838.1	Chimpanzee	838	25.78	22.91	41.53

### Appendix B.4. Set D

The sequences in this set correspond to the TREM2 protein (Triggering Receptor Expressed on Myeloid cells 2) in different species: human, mouse, rat, and baboon. TREM2 is a receptor expressed primarily on immune system cells, such as microglia in the brain. In older adults, mutations or dysfunction in TREM2 have been associated with an increased risk of neurodegenerative diseases, especially Alzheimer’s. Table A4 details the characteristics of the sequences in Set D.

**Table A4:** Description of the sequences in Set D.

Accession	Species	Length (aa)	Polar Residues (%)	Hydrophobicity (GRAVY)
AAH32362.1	Homo sapiens	222	51.4	−0.468
Q99NH8.1	Mus musculus	227	44.5	−0.03
NP_001418899.1	Rattus norvegicus	229	47.6	−0.016
XP_011842060.1	Mandrillus leucophaeus	221	51.6	−0.464
